# Identifying key antioxidative stress factors regulating Nrf2 in the genioglossus with human umbilical cord mesenchymal stem-cell therapy

**DOI:** 10.1038/s41598-024-55103-8

**Published:** 2024-03-10

**Authors:** Haixian Guo, Yue Liu, Xinlu Yu, Na Tian, Yan Liu, Dan Yu

**Affiliations:** 1 Department of Otolaryngology Head and Neck Surgery, The Second Hospital of Jilin University, 4026 Yatai Street, Changchun, 130041 Jilin Province China; 2 Jilin Tuohua Biotechnology Co., LTD, Tiedong District, Siping, 136000 Jilin Province China

**Keywords:** Molecular biology, Stem cells, Diseases, Medical research, Molecular medicine

## Abstract

Intermittent hypoxia in patients with obstructive sleep apnea (OSA) hypopnea syndrome (OSAHS) is associated with pharyngeal cavity collapse during sleep. The effect of human umbilical cord mesenchymal stem cells (HUCMSCs) on OSA-induced oxidative damage in the genioglossus and whether nuclear factor erythroid 2-related factor 2 (Nrf2) or its upstream genes play a key role in this process remains unclear. This study aimed to identify the key factors responsible for oxidative damage during OSAHS through Nrf2 analysis and hypothesize the mechanism of HUCMSC therapy. We simulated OSA using an intermittent hypoxia model, observed the oxidative damage in the genioglossus and changes in Nrf2 expression during intermittent hypoxia, and administered HUCMSCs therapy. Nrf2 initially increased, then decreased, aggravating the oxidative damage in the genioglossus; Nrf2 protein content decreased during hypoxia. Using transcriptomics, we identified seven possible factors in HUCMSCs involved in ameliorating oxidative stress by Nrf2, of which DJ-1 and MEF2A, showing trends similar to Nrf2, were selected by polymerase chain reaction. HUCMSCs may reduce oxidative stress induced by intermittent hypoxia through Nrf2, and the possible upstream target genes in this process are MEF2A and DJ-1. Further studies are needed to verify these findings.

## Introduction

Obstructive sleep apnea (OSA) and hypopnea syndrome (OSAHS) is a type of sleep disorder caused by recurrent, complete, or incomplete obstruction of the upper airway. This leads to intermittent hypoxia or sleep-disordered breathing accompanied by hypercapnia^1^, can further lead to formation of lesions, affecting quality of life, and may endanger the life of patients in severe cases.

Imageological examination studies have determined that pharyngeal anatomical abnormalities in OSAHS patients are related to pharyngeal cavity collapse during sleep^2^. The upper airway is a highly malleable structure that lacks bone support and is susceptible to collapse force. For example, the intraluminal pressure generated during inspiration and the extraluminal pressure exerted by the surrounding soft tissues are both important components of collapse force and may be primarily responsible for OSAHS. The upper airway dilator muscles include the genioglossus, sternohyosus, and geniohyoideus, with the genioglossus referred to as the “safety muscle” owing to its importance. Insufficient activity of the genioglossus may lead to upper airway collapse caused by negative inspiratory pressure during sleep, causing hypopnea or apnea, and eventually leading to development of OSAHS. An earlier study revealed a high correlation between genioglossus activation and upper airway negative pressure, and showed increase in genioglossus activity under hypoxic and high carbonic acid conditions, leading to fatigue^3^. Simultaneously, hypoxia associated with OSAHS may cause damage to the genioglossus, manifesting as increased muscle fatigue, further aggravating the OSAHS, thereby forming a vicious circle.

Research has indicated that chronic intermittent hypoxia (CIH) can enhance the expression of reduced coenzyme II oxidase in the genioglossus and elevate oxidative stress levels^4^. Empirical investigations have demonstrated that individuals with moderate and severe OSAHS exhibit significantly higher levels of serum markers of oxidative stress compared to healthy individuals, with the severity of OSAHS being positively associated with oxidative stress. Conversely, continuous positive airway pressure effectively ameliorates ventilation and hypoxia, thereby preventing the onset of oxidative stress^5^.

Nuclear factor erythroid 2-related factor 2 (Nrf2) is an important regulator of antioxidant damage. Under normal circumstances, Nrf2 is mainly inhibited by Kelch sample-related protein-1 (epoxy chloropropane Kelch sample-related protein -1, KEAP1). KEAP1 is bound and located in the cytoplasm. In the event of increased oxidative stress, Nrf2 dissociates from KEAP1 and enters the nucleus to stimulate an increase in the expression of downstream antioxidant genes, thus playing a role in preventing oxidative damage^6,7^. The major downstream antioxidant genes stimulated by Nrf2 are NAD(P)H quinone oxidoreductase 1 (NQO-1), Heme oxygenase 1 (HO-1) and Glutathione S transferase-alpha 1 (GST-α1)^8^. The protein activity of Nrf2 can be further defined by detecting the expression of its downstream genes.

Studies have shown that during cardiac injury caused by pathological conditions closely related to oxidative stress, such as hypertension, diabetes, and ischemia reperfusion, Nrf2 increased in the early stage and gradually decreased in the late stage. Furthermore, the increased expression of Nrf2 has an obvious protective effect on cardiac injury^9–12^. Currently, it is unknown whether Nrf2 has a protective effect on a mentoglossus injury caused by CIH.

Human umbilical cord mesenchymal stem cells (HUCMSCs) are important members of the stem cell family, having multi-differentiation potential, immune regulation, and self-replication characteristics. In vivo, HUCMSCs can chemotax, homing to damaged tissues and promoting tissue repair or regeneration. Since HUCMSCs originate from the early development of the mesoderm, their immunogenicity is low, and their surface antigens are not prominent, thereby preventing any major allograft rejection. HUCMSCs have greater proliferation and differentiation ability than mesenchymal stem cells (MSCs) from the bone marrow^13^. The application of HUCMSCs is not limited to age, the source is broad, and there is no adverse effect on the donor. Further, there are no ethical or moral constraints. Therefore, HUCMSCs have broad application prospects.

Some authors have studied the potential role of HUCMSCs in OSA using an acute rat model simulating recurrent airway obstruction of OSA, suggesting that HUCMSCs may act as a regulator of inflammation and repair tissue damage induced by OSA^14^. HUCMSCs are currently used to treat many diseases, such as hematopoietic stem cell transplantation, tissue damage repair, and autoimmune diseases.

Various signaling pathways are involved in the protective effect of MSCs against oxidative damage. The Nrf2/HO-1 signaling pathway is one of the major signaling pathways. MSCs can reduce isopropanol-induced lung injury and cell apoptosis by up-regulating Nrf2 expression^15^. The pathway is activated by MSCs and has an anti-apoptotic protective effect on spinal cord injury^16^. HUCMSC transplantation can increase the expression level of the Nrf2 gene in the injured liver of mice^17^. However, the effect of HUCMSCs on oxidative damage in genioglossus cells in an OSA model and whether Nrf2 plays a key role in this process remain unclear. Therefore, this study aimed to identify the key factors responsible for oxidative damage during OSAHS through Nrf2 and hypothesize the mechanisms of MSC therapy in ameliorating oxidative damage in the genioglossus.

## Results

**Figure 1:**
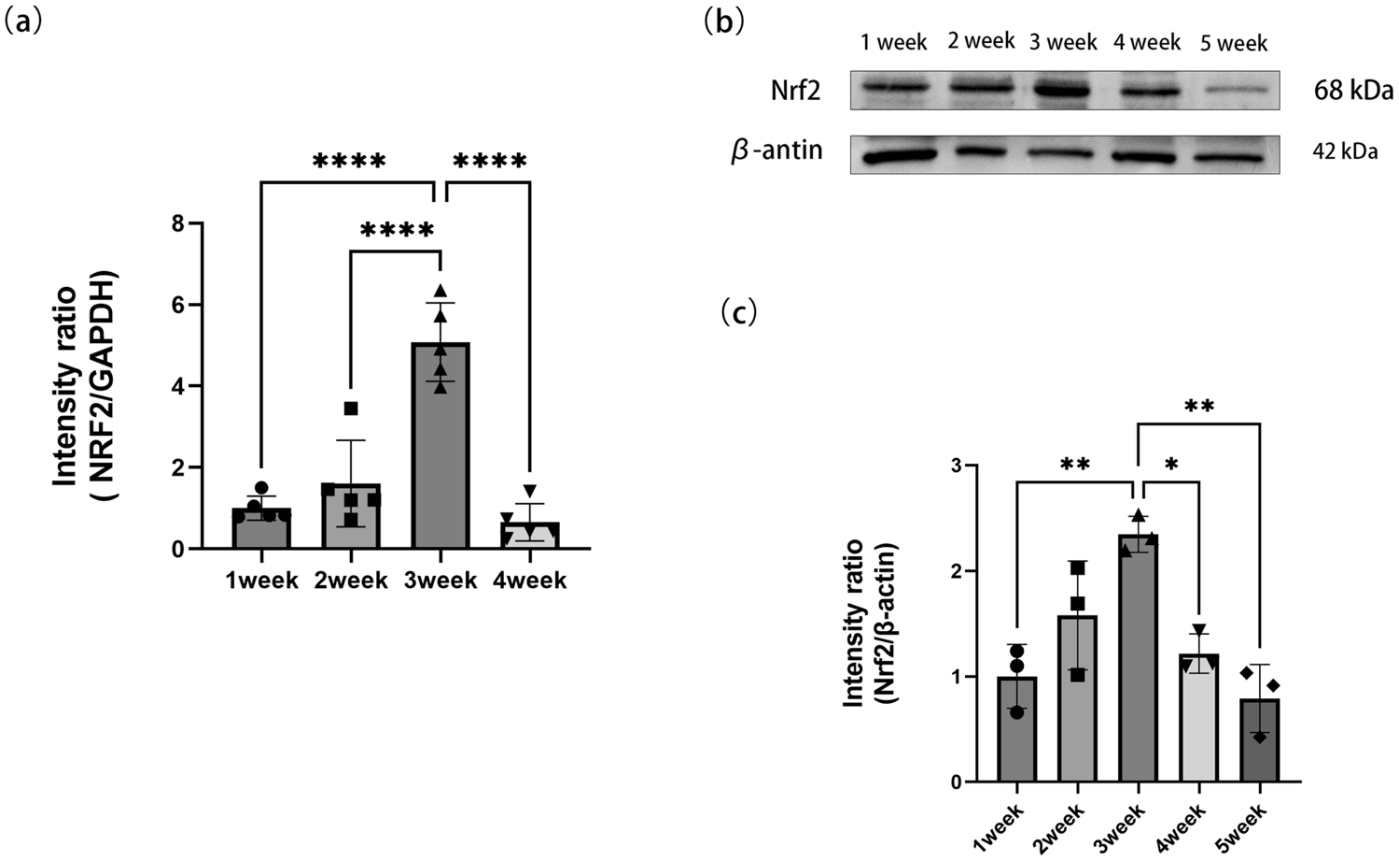
Relative expression of Nrf2. (**a**) qRTPCR was used to measure the expression of Nrf2 in genioglossus intermittent hypoxia sampled weekly. (**b, c**) Western blot was used to measure the expression of Nrf2 in genioglossus intermittent hypoxia sampled weekly. *p < 0.05; **p < 0.01; ***p < 0.001; n ≥ 3.

We simulated moderate to severe OSA in an animal model with intermittent hypoxia. The RNA expression and protein content of Nrf2 in the genioglossus sampled weekly is shown in Fig. 1.

Nrf2 in the genioglossal muscle increased gradually during the first 3 weeks of CIH and decreased in the 4th week. We speculate that the initial increase in Nrf2 in the genioglossus resulted in compensatory improvement in the antioxidant defense ability, and decompensated in the late stage with a decrease in antioxidant defense ability.

### Determination of MDA content and GSH-PX activity

**Figure 2:**
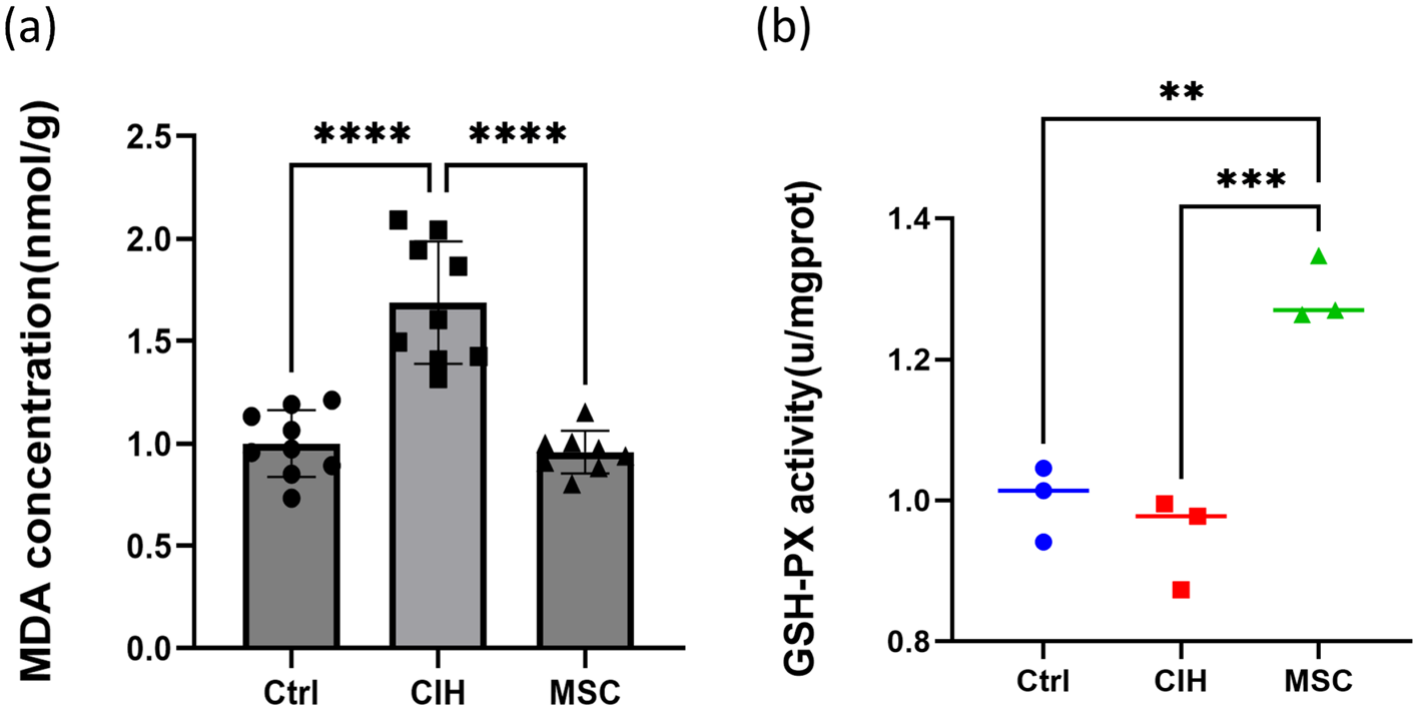
The content of MDA and GSH-PX activity. (**a**) The content of MDA in genioglossus, (**b**) GSH-PX activity. Ctrl, control group. CIH, intermittent hypoxia group. MSC, MSC therapy group. n ≥ 3, *p < 0.05; **p < 0.01; ***p < 0.001.

At the 5th week of CIH, when Nrf2 levels began to decline, HUCMSC therapy was administered, the malondialdehyde (MDA) content and GSH-PX activity in the genioglossus was measured. Figure 2 shows that MDA content in the CIH group was higher than that in the control group, and MDA content in the HUCMSCs group was lower than that in the CIH group. GSH-PX activity in the CIH group was lower than that in the control group, and in the HUCMSCs group was higher than that in the CIH group. MDA content can indirectly reflect the degree of tissue peroxidation damage. GSH-Px is an important peroxide breaking enzyme that exists widely in the body. It can reflect the ability of the body to resist oxidative damage. From these results, intermittent hypoxia may aggravate the oxidative stress injury of the genioglossus, and the administration of HUCMSCs therapy can alleviate this oxidative damage and increase the ability to resist oxidative damage.

**Figure 3:**
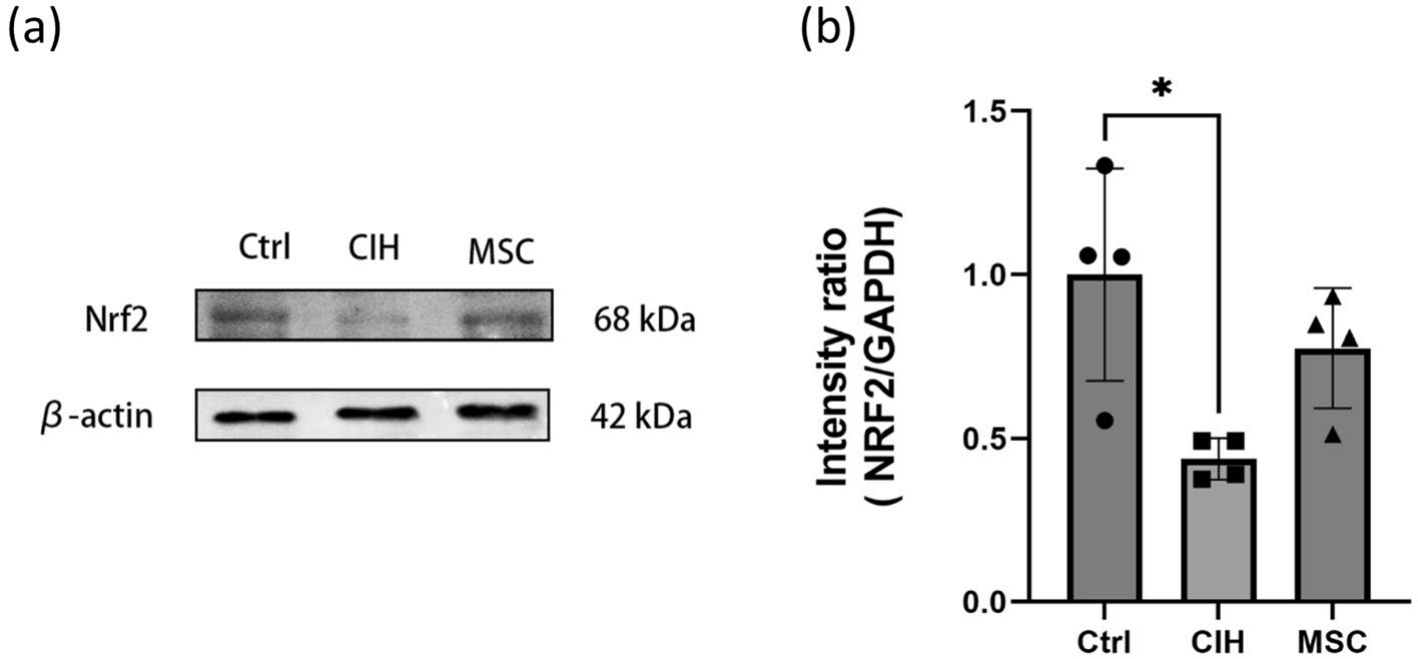
The relative protein expression of Nrf2 in genioglossus. (**a**, **b**) The protein content of Nrf2 in the genioglossus. Ctrl, control group. CIH, intermittent hypoxia group. MSC, MSC therapy group. n ≥ 4, *p < 0.05.

**Figure 4:**
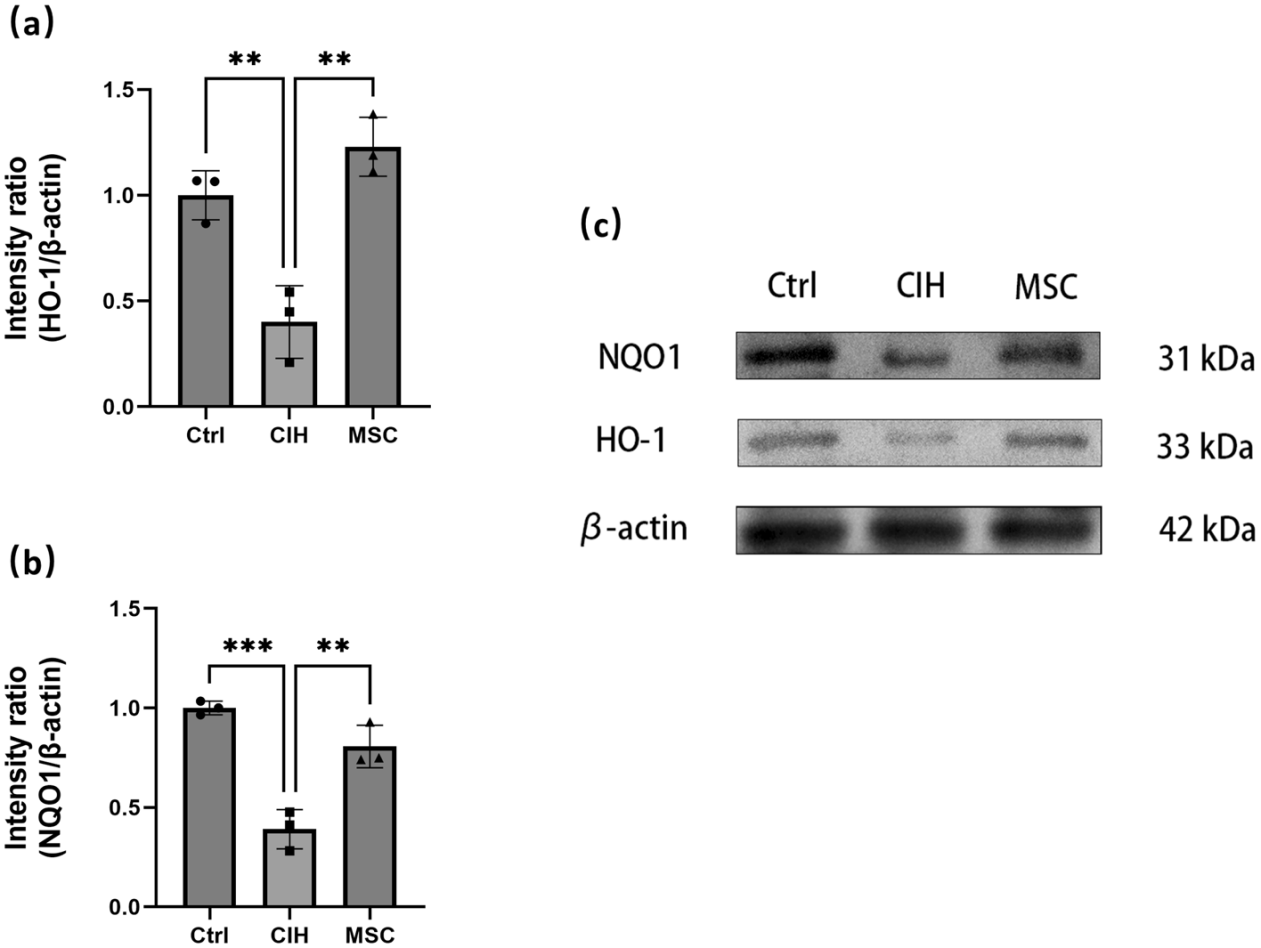
The protein content of NRF2 downstream genes HO-1 NQO1 in genioglossus. (**a, c**) Western blot was used to measure the expression of NRF2 downstream genes HO-1 in the genioglossus. (**b**, **c**) Western blot was used to measure the expression of NRF2 downstream genes NQO1 in the genioglossus. Ctrl, control group. CIH, intermittent hypoxia group. MSC, MSC therapy group. n ≥ 3, *p < 0.05; **p < 0.01; ***p < 0.001.

To assess whether HUCMSCs can decrease oxidative stress injury through Nrf2 regulation, protein content of Nrf2 along with protein content of downstream genes HO-1 and NQO1 in the genioglossus were measured, respectively, and the results are shown in Figs. 3 and 4.

The protein content of Nrf2 and protein content of downstream genes HO-1 and NQO1 in the genioglossus of the CIH group were lower than that of the control group, while in the MSC group, they were higher than that in the CIH group. HUCMSCs may reduce the oxidative damage caused by intermittent hypoxia and increase the ability to resist oxidative damage by increasing the expression of Nrf2 and its downstream genes HO-1 and NQO1 in the genioglossus.

**Figure 5:**
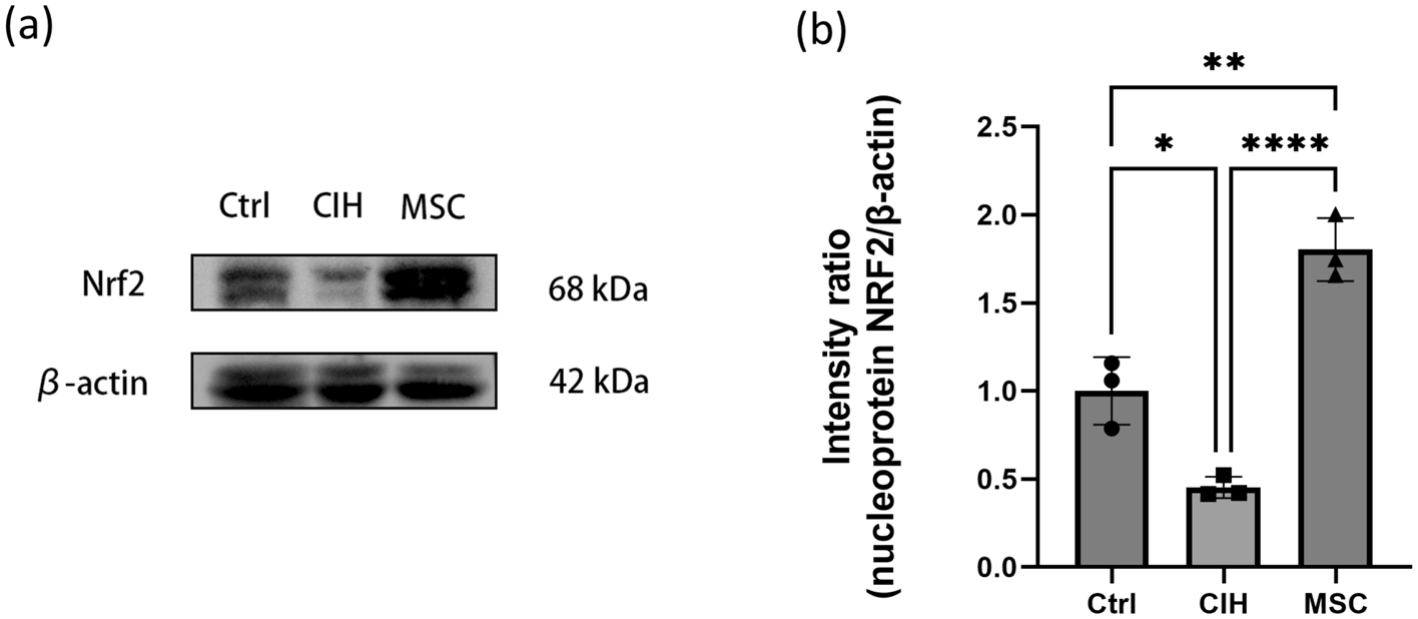
The relative nucleoprotein expression of Nrf2 in genioglossus. (**a**, **b**) The nucleoprotein content of Nrf2 in the genioglossus. Ctrl, control group. CIH, intermittent hypoxia group. MSC, MSC therapy group. n ≥ 3, *p < 0.05.

To explore the possible ways in which hUCMSCs promote Nrf2 expression, nucleoprotein content of Nrf2 was measured, and the results are shown in Fig. 5.

The nucleoprotein content of Nrf2 in the genioglossus of the CIH group were lower than that of the control group, while in the MSC group, they were higher than that in the CIH group. HUCMSCs may reduce the oxidative damage caused by intermittent hypoxia and increase the ability to resist oxidative damage by promotes nucleus displacement of NRF2.

### Transcriptomics

Nrf2 is a classic antioxidant-regulating factor against oxidative stress. To determine whether MSCs can activate Nrf2 by regulating other factors, we used transcriptomics to search for possible upstream factors of HUCMSCs ameliorating oxidative stress through Nrf2.

**Figure 6:**
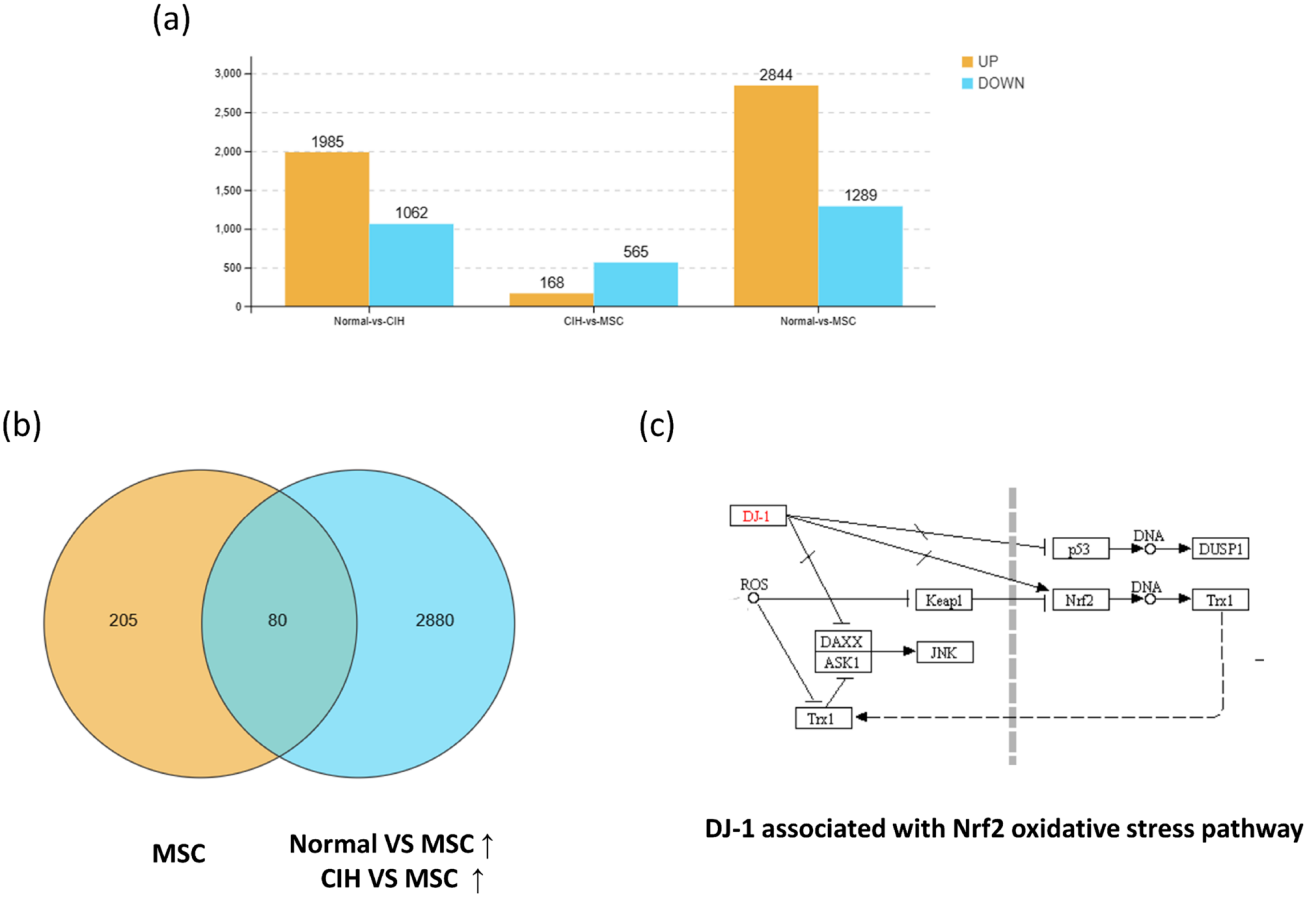
Transcriptomics. (**a**) Different groups of up-regulated and down-regulated genes. (**b**) 285 genes unique to MSC, 2960 genes up-regulated by CIH vs MSC and Control vs MSC, and 80 genes in the two gene sets intersected. (**c**) DJ-1 gene directly associated with Nrf2 oxidative stress pathway (Ko05012).

**Figure 7:**
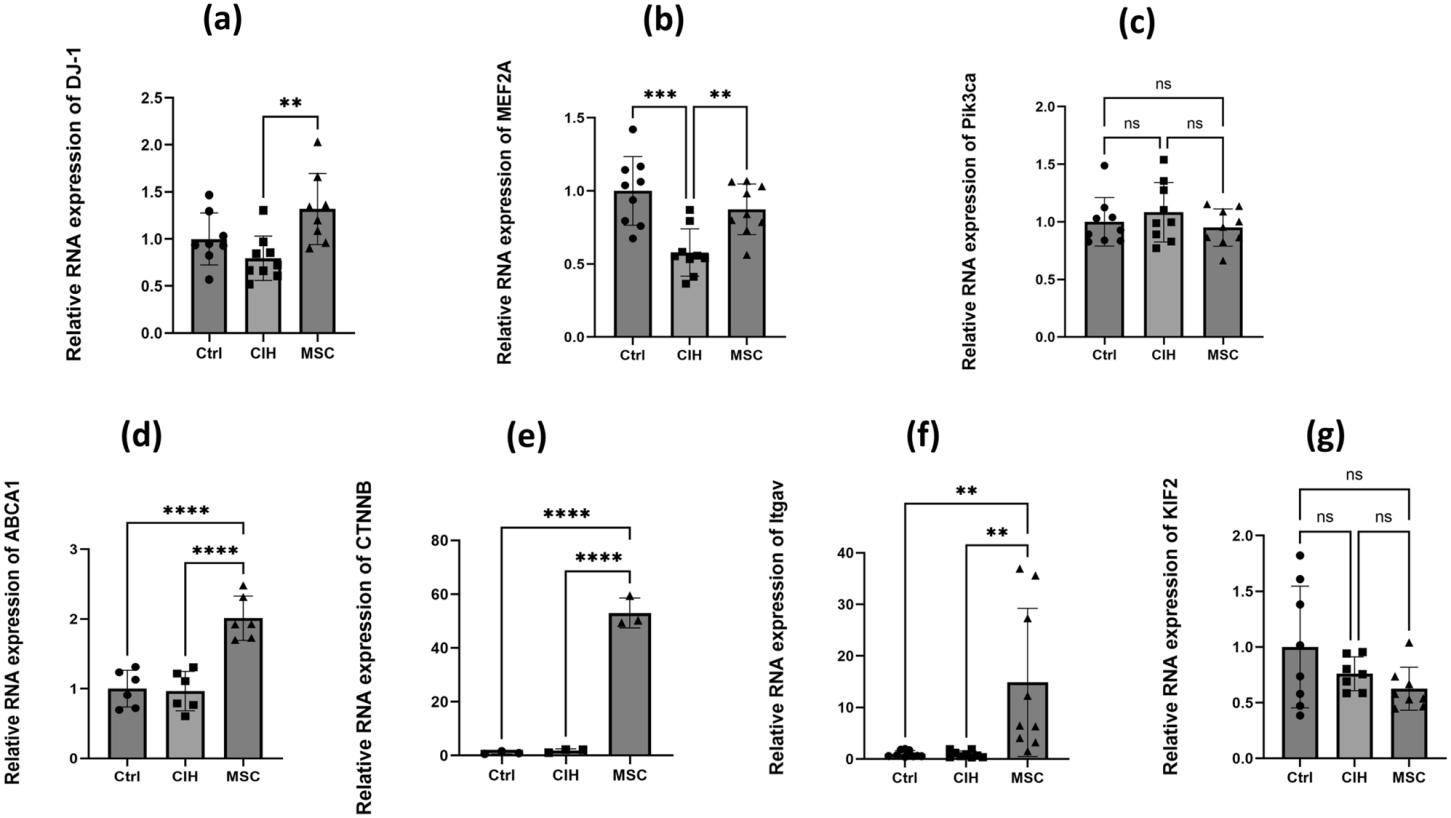
Relative RNA expression of the 7 genes. (**a**) Relative RNA expression of DJ-1. (**b**) Relative RNA expression of Mef2a. (**c**) Relative RNA expression of Pik3ca. (**d**) Relative RNA expression of ABCA1. (**e**) Relative RNA expression of CTNNB. (**f**) Relative RNA expression of Itgav. (**g**) Relative RNA expression of KIF2. Ctrl, control group. CIH, intermittent hypoxia group. MSC, MSC therapy group. n ≥ 3, *p < 0.05; **p < 0.01; ***p < 0.001.

There were 285 genes unique to MSCs, 2960 genes up-regulated by CIH vs. MSC and control vs. MSC, and 80 genes in the two gene sets intersected. Seven pathways were associated with Nrf2 and were marked in red or red and green boxes, and correlation analysis was conducted with Nrf2 (Fig. 6). The top 10 genes with Pearson coefficient > 0.8 were screened out, five of which were directly related to the Nrf2 oxidative stress pathway;two genes directly associated with Nrf2 oxidative stress in seven (Table 1). Nrf2-related pathways were screened out for PCR verification (Table 2) (Fig. 7).

**Table 1:** Seven genes directly associated with Nrf2 oxidative stress pathway.

ID	Symbol	Pearson	P	Pathway ID associated with Nrf2
ENSRNOG00000056371	Pik3ca	0.960530116	< 0.05	Ko05418/Ko05200/Ko05225/Ko04141 Ko05417/Ko05012/Ko05208
ENSRNOG00000047756	Mef2a	0.921843847	< 0.05	Ko05418
ENSRNOG00000004912	Itgav	0.830929014	< 0.05	Ko05200/Ko05418
ENSRNOG00000054172	CTNNB	0.822068165	< 0.05	Ko05200/Ko05418
ENSRNOG00000067705	KIF2	0.675609049	< 0.05	Ko05418
ENSRNOG00000018289	DJ-1(PARK7)	0.632656833	0.0546897441	Ko05012
ENSRNOG00000018126	ABCA1	0.07021936	0.857536778	kO05417

**Table 2:** Nucleotide sequences of primers used for PCR amplification.

Genes	Forward primer	Reverse primer
Nrf2	5′-CACATCCAG TCAGAAACCAGTGG-3′	5′-GGAATGTCTGC GCCAAAAGCTG-3′
DJ-1	5′-TTCCTGTGGATGTCATGCGG-3′	5′-CTTTGAGAACAAGCGGTGCC-3′
Metf2a	5′-CACACGCATAATGGATGAGAGG-3′	5′-ATATCCGAGT TCGTCCTGCT T-3′
Pi3k2a	5′-CATTGCCCCTCCTGATGT-3′	5′-GGTGCTGGCTGTCTCTCATT-3′
ABCA1	5′-GCTGGTGTGGACCCT TACTC-3′	5′-AAATGCCCAGGTCTGAGAGC-3′
CTNNB	5′-TTGAAGGT TGTACCGGAGCC-3′	5′-GCCACCCATCTCATGT TCCA-3′
Itgav	5′-GGTCCCCAAGTCACTCCAAG-3′	5′-CTGCTGGTGCACACTGAAAC-3′
KIF2	5′-CTTCATCCTGTCCATGGGGC-3′	5′-CGTCGTCGAAGAGACCGAA-3′

**Figure 8:**
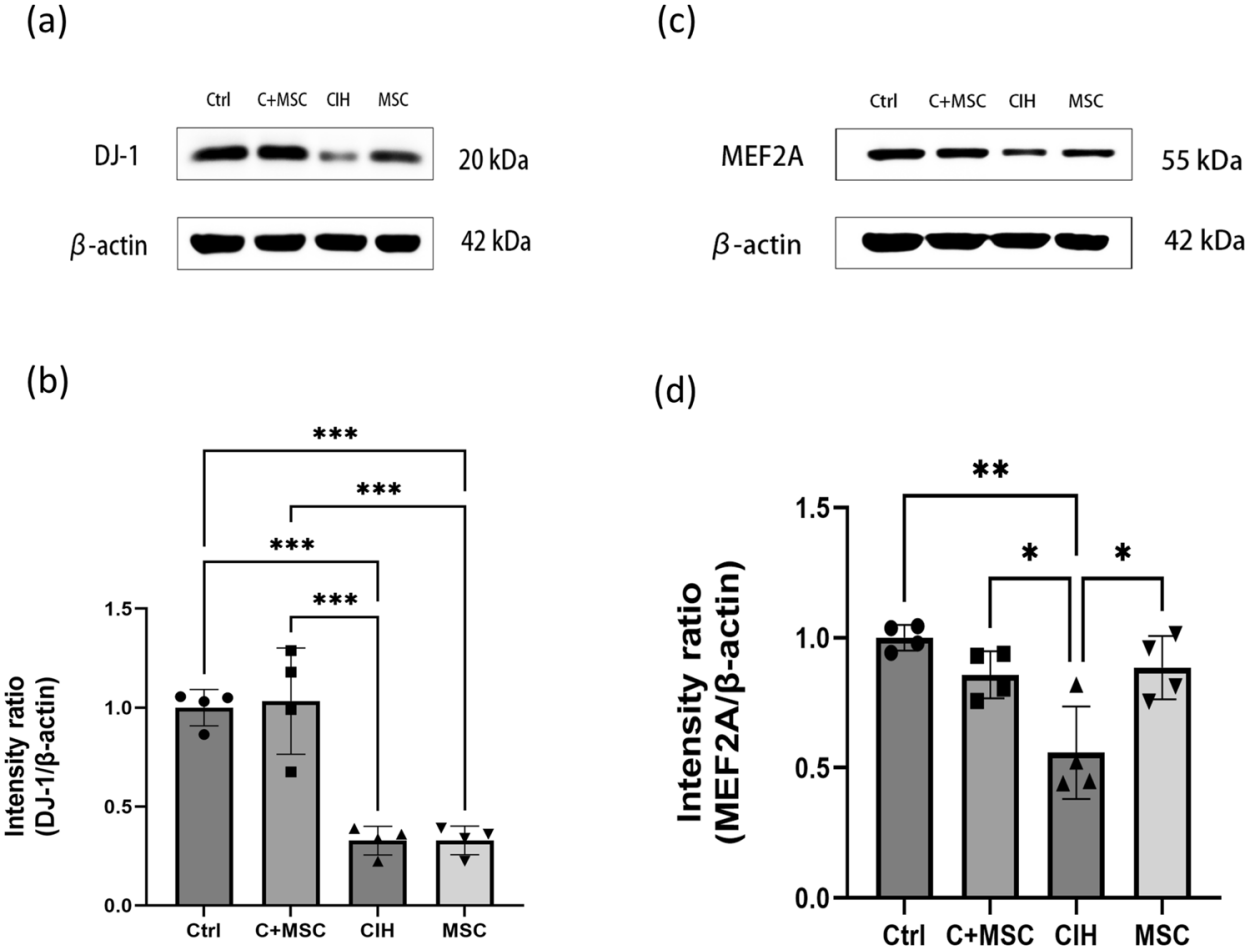
The protein content of DJ-1 and MEF2A in genioglossus. (**a**, **b**) The protein content of DJ-1 in the genioglossus. (**c**, **d**) The protein content of MEF2A in the genioglossus. Ctrl, control group. C + MSC, control group + MSC therapy. CIH, intermittent hypoxia group. MSC, MSC therapy group. n ≥ 4, *p < 0.05; **p < 0.01; ***p < 0.001.

The mRNA expressions of MEF2A and DJ-1 decreased in the CIH group compared with the control group and increased in the MSCs compared with the CIH group; the changes found were similar to that of Nrf2. The protein contents of MEF2A and DJ-1 were similar to that of Nrf2 as well (Fig. 8). The protein contents of MEF2A and DJ-1 decreased in the CIH group compared with the control group and increased in the MSC compared with the CIH group. MEF2A and DJ-1 may therefore be the key factors causing the increase of Nrf2 and reducing the oxidative stress damage caused by intermittent hypoxia during the treatment with HUCMSCs. The gene knock-down of MEF2A and DJ-1 was performed respectively to further verify the relationship between the above factors and Nrf2.

## Discussion

In this study, we simulated moderate to severe OSAHS in an animal model with intermittent hypoxia and measured the gene expression and protein content of Nrf2 in rat genioglossus tissues weekly. We found that Nrf2 increased in the early stage and decreased in the late stage, conjecturting that the antioxidant capacity of genioglossus increased in the early stage and later reduced in a hypoxic environment. In the early stage^18^, the research group has confirmed that the early intervention of HUCMSCs can alleviate the genioglossus injury caused by OSAHS, and the late treatment can reverse the decline of the structure and function of the genioglossus caused by OSAHS to a certain extent. When Nrf2 decreased, HUCMSC treatment was given, MDA content, GSH-PX activity along with Nrf2, HO-1, and NQO1 protein levels, Nrf2 nucleoprotein level were measured. The oxidative damage in the genioglossus was aggravated, Nrf2 protein and nuleoprotein contents decreased during hypoxia. After MSC treatment, oxidative damage was alleviated, the ability to resist oxidative damage increased, and Nrf2, HO-1, and NQO1 protein levels increased, Nrf2 nuleoprotein level increased,suggesting that HUCMSCs may play an antioxidant role through facilitate Nrf2 enters the nucleus and stimulate an increase in the expression of downstream antioxidant genes NQO1 and HO-1, increase GSH-PX activity. Nrf2 is a classic antioxidant-regulating factor against oxidative stress. Through transcriptomics, we found seven possible factors related to the reduction of oxidative stress by Nrf2 by HUCMSCs, screened them via polymerase chain reaction (PCR), and selected two factors, DJ-1 and MEF2A, with expression trends similar to Nrf2. We speculate that MEF2A and DJ-1 may be the key factors that cause the increase of Nrf2 and reduce the oxidative stress damage, increase the ability to resist oxidative damage caused by intermittent hypoxia during treatment with HUCMSCs.

OSAHS-associated oxidative stress is caused by an increase in the pro-oxidation/antioxidant ratio, mainly due to the production of hypoxia during apnea events and the formation of reactive oxygen species (ROS) during re-oxygenation in respiratory recovery^19^. Nrf2 is up-regulated in response to ROS elevation, participating in protective mechanisms to counteract certain harmful effects of ROS^20^. Studies have shown that intermittent hypoxia can improve the anti-oxidative stress ability of rat lung tissue by promoting Nrf2 intracellular displacement and activating Nrf2 in the early stage^21^. Studies have also shown that oxidative stress induced by intermittent hypoxic exposure plays a crucial role in the pathogenesis of tumor metastasis, and the administration of antioxidants may be a unique strategy for patients with OSAHS-associated cancer^22^. These results may have important implications for the diagnosis and treatment of OSA.

Nrf2 is an important regulator of cellular endogenous antioxidant response and can induce key genes involved in antioxidant reactions^23^. Oxidative stress has been shown to activate Nrf2 gene expression and transcriptional activity in vitro (C2C12 skeletal muscle cells) and in vivo (rodent muscles)^24–27^. Nrf2 regulates the expression of antioxidant genes by interacting with antioxidant response elements(ARE) and regulating oxidative stress response^28^.

NRF2 is an unstable, short-lived protein under unstressed conditions, it is principally controlled by Kelch-like ECH-associated protein 1 (KEAP1)^29,30^. KEAP1 prevent NRF2 for degradation, leading to NRF2 accumulation. KEAP1-Nrf2 located in the cytoplasm.Oxidative stress prompt Nrf2 dissociates from KEAP1 and enters the nucleus to stimulate an increase in the expression of downstream antioxidant genes, thus playing a role in preventing oxidative damage^6,7^. That is why Nrf2 in the genioglossal muscle increased gradually during the first 3 weeks. Conjecture with the consumption of dissociated Nrf2, the protein content of Nrf2 gradually decreased, and the antioxidant effect mediated by Nrf2 gradually weakened, resulting in increased oxidative stress injury. Nrf2 in the genioglossal muscle decreased in the 4th week. In the 5th week, Nrf2 content continued to decrease, MDA content increased, and oxidative stress injury increased.

DJ-1 (also known as PARK7) is an autosomal recessive gene associated with Parkinson's disease that has multiple functions^31^, including active ROS scavenging, and is an important regulator of antioxidant gene induction. The subcell of DJ-1 is located in the mitochondrial matrix and inner membrane and is involved in the physiological function of the mitochondria, protecting it against oxidative stress^32,33^. Several studies have shown that DJ-1 has a clear anti-oxidative stress effect and an upstream and downstream relationship with Nrf2. DJ-1 can promote the nuclear translocation of Nrf2 through PI3K/AKT/GSK-3β signal transduction. Thus, the expression of downstream antioxidant enzymes such as heme oxygenase-1 (HO-1), NAD(P)H quinone oxidoreductase 1 (NQO-1), and other Nrf2-related antioxidant enzymes, including SOD1, peroxidase reductase, and thioredoxin reductase increased^34^. DJ-1 also enhances mitochondrial isocitrate dehydrogenase expression through the KEAP1/Nrf2 pathway, thereby preventing oxidative stress and neurodegeneration^35^. DJ-1 can indirectly stabilize the expression level of Nrf2, the master regulator of antioxidant transcription, by preventing KEAP1 from binding to Nrf2^36,37^. The severity of oxidative stress in cells or tissues is determined by the production and removal of oxides. DJ-1 can protect cells from oxidative stress-induced cell death^36,38^.

The transcription factor MADS family (MCM1, Agamous, defens, and SRF) includes four MEF2 proteins, myocyte enhancer factor 2A (MEF2A) to MEF2Dl^39^. MEF2A is a widely distributed DNA-binding transcription factor. A study has shown that MEF2A can inhibit cell senescence by positively regulating the PI3K/Akt signaling pathway^40^. Mir-615-3p inactivates the P13K/Akt pathway by inhibiting MEF2A, thereby reducing oxidative stress damage^41^. However, studies have also shown that gene p66shc promotes cellular oxidative stress by simultaneously inhibiting MEF2A expression and KLF2 transcription^42^. MEF2A plays varied roles in different aspects of anti-oxidative stress mechanisms.

**Figure 9:**
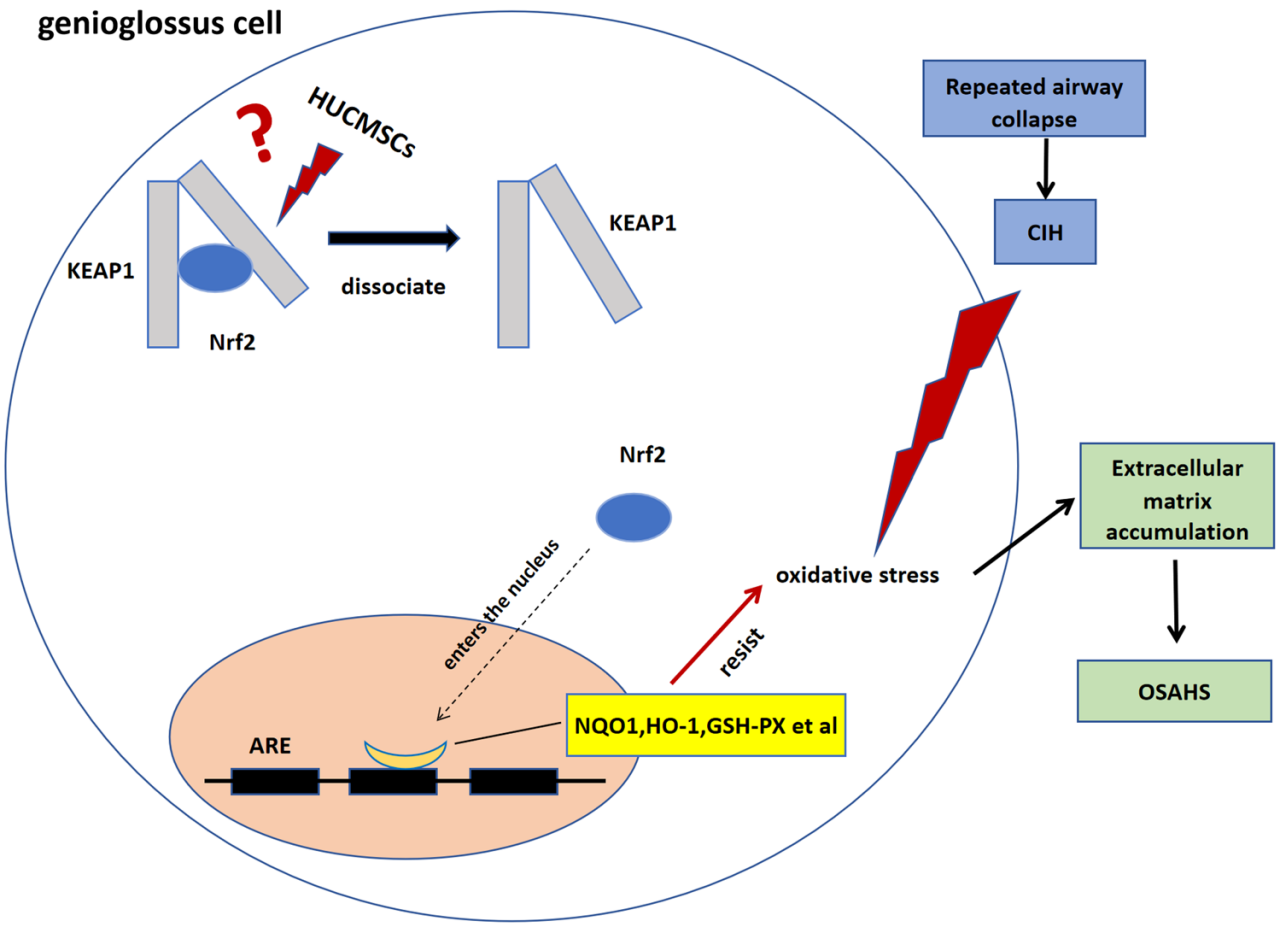
Diagram of the possible mechanism of HUCMSCs improvement oxidative stress through NRF2. HUCMSCs, human umbilical cord mesenchymal stem cells (HUCMSCs); KEAP1, Kelch sample-related protein-1; Nrf2, nuclear factor erythroid 2-related factor 2; ARE, antioxidant response element; NQO1, NAD(P)H quinone oxidoreductase 1; HO-1, heme oxygenase 1; GSH-PX, glutathione peroxidase; CIH, chronic intermittent hypoxia.

The possible mechanism of HUCMSCs improvement oxidative stress through NRF2 is when the upper airway is repeatedly collapse, chronic intermittent hypoxia acts on the genioglossus, leading to the occurrence and progression of OSAHS through oxidative damage. We speculate that HUCMSCs may be directly or indirectly enable DJ-1/MEF2A, make Nrf2 dissociates from KEAP1 and enters the nucleus to stimulate an increase in the expression of downstream antioxidant genes. The major downstream antioxidant genes stimulated by Nrf2 are NQO1 and HO-1. GSH-PX activity increased. Thus plays a role in preventing oxidative damage and delay the occurrence and development of OSAHS (Fig. 9).

The limitations of this study are the small sample size and the lack of further confirmatory in vitro cell experiments. in a subsequent study, in vitro cell assays with MEF2A and DJ-1 gene knock-downs will be performed, respectively, to verify possible pathway mechanism of HUCMSCs improvement antioxidative stress through NRF2.

## Materials and methods

### Ethical approval

This study is reported in accordance with ARRIVE guidelines. This study was approved by the Animal Ethics Committee of Changchun Weishi Testing Technology Service, People’s Republic of China (approval number: 20220906-01), and all the animal care and experimental procedures were conducted in accordance with “The Guide for Care and Use of Laboratory Animals.”

### Experimental grouping and establishment of intermittent hypoxic animal model

Thirty SPF Wistar rats, aged 4 weeks and weighing 200–300 g, were divided into control, CIH, and MSC treatment groups. In the early stage^43^, the research group put the rats into the intermittent hypoxic animal chamber (intermittent hypoxic chamber XF-2CL, Nanjing Xinfei Instrument Manufacturing Co., LTD.) and the same conditions in the normal oxygen test chamber. Intermittent hypoxia animal chamber establishes an intermittent hypoxia mode with oxygen concentration set at 10–21% and 120 s for one cycle by setting the time and flow of oxygen and nitrogen supply. At the same time, the oxygen detector (Xima AR8100) is placed in the animal compartment to monitor the oxygen changes in the animal compartment. First inject nitrogen to make the minimum oxygen concentration in the cabin reach 7 ± 0.5%, it takes about 45–50 s, and then inject oxygen to restore the oxygen concentration in the cabin to 21 ± 0.5%, ensuring that the concentration in the cabin is repeated between 7 and 21%. The oxygen concentration in the normoxic chamber is 21%. Arterial oxygen saturation (SaO2) was monitored. During the 120 s period, the oxygen concentration in the intermittent hypoxia chamber first decreased and then increased, and the arterial oxygen saturation (SaO2) of rats also showed a trend of first decreasing and then increasing, which could simulate the pathogenic process of OSAHS. In the intermittent hypoxia environment, the lowest arterial oxygen saturation of rats was 59.17%, and that of the normal oxygen control group was 94.33–97.33%. Intermittent hypoxia chamber set oxygen concentration of 10–21%, 120 s a cycle, can simulate moderate and severe OSAHS.

The CIH and MSC groups were placed in the intermittent hypoxia chamber, with oxygen concentration set at 10 − 21% and the cycle time at 120 s. The control group was placed in the normal oxygen test chamber. They were placed in a hypoxic chamber and a control chamber for 8 h a day (08:00 am–16:00 pm) for 5 weeks. The experimental protocols in the present study including all the surgical procedures and animal usages were in line with the ARRIVE guidelines and approved by the Animal Ethics Committee of Changchun Weishi Testing Technology Service .

### Treatment

After 5 weeks (when Nrf2 levels began to decline), the control group was injected with 1 ml of normal saline in the tail vein, and the treatment group was injected with HUCMSCs (provided by Jilin Tuohua Biotechnology Co., LTD., which has been certified by the State Food and Drug Administration Institute), 2 × 10^6^ cells/ml, 1 ml, once a week, for four weeks.

### Transcriptomics

Total RNA was extracted using a TRIzol reagent kit (Invitrogen, Carlsbad, CA, USA) according to the manufacturer’s protocol. RNA quality was assessed on an Agilent 2100 Bioanalyzer (Agilent Technologies, Palo Alto, CA, USA) and checked using RNase-free agarose gel electrophoresis. After total RNA was extracted, eukaryotic mRNA was enriched by Oligo (dT) beads. The enriched mRNA was then fragmented using fragmentation buffer and reverse transcribed into cDNA using the NEBNext Ultra RNA Library Prep Kit for Illumina (NEB #7530, New England Biolabs, Ipswich, MA, USA). The purified double-stranded cDNA fragments were end-repaired, a base added, and ligated to Illumina sequencing adapters. The ligation reaction was purified with the AMPure XP Beads (1.0 ×) and PCR amplified. The resulting cDNA library was sequenced using Illumina Novaseq6000 by Gene Denovo Biotechnology Co. (Guangzhou, China). The animal model of intermittent hypoxia was established, and three genioglossus samples were taken from the control, CIH, and MSC treatment groups. RNA-seq analysis was performed based on the criterion of a fold change ≥ 1.5, false discovery rate ≤ 0.05.

### Experimental method

#### Protein concentration detection and Western blot

For western blotting, 30 mg of sample was mixed with 300 μl of RIPA lysis bufer (Yuanye Biotechnology Co., Ltd., Shanghai, China) and PMSF (100 mM) (BL507a, biosharp, China). Then, samples were centrifuged at 12,000 rpm and 4 °C for 15 min. The protein concentration of each protein lysate (the supernatant) was then determined with a BCA Protein Assay Kit (BL1054S, biosharp, China). Then, we took the same protein content from each sample (50–100 μg) and added the same volume of 5× protein buffer (p1041, Solarbio Science & Technology Co., Ltd., Beijing, China). Then, protein samples were boiled for 5 min in boiling water. Exactly the same protein content was loaded on a 10% SDS-PAGE (P0012A, Beyotime Biotechnology Co., Ltd., Shanghai, China), separated proteins were then transferred to PVDF membranes and cut the membranes into suitable size according to protein ladder and the molecular weight of target protein.. Membranes were then blocked with 5% non-fat dry milk for 1 h at room temperature. The target proteins were detected with primary antibodies diluted 1:5000–1:200 in phosphate-buffered saline with Tween® (PBST) at 4 °C overnight. Antibodies against β‑actin (R1102-1; Hangzhou Huaan Biotechnology Co., LTD.), Nrf2 (No. 16396-1-AP, Proteintech), NQO1 (T56710, Abmart), HO-1 (TA5393, Abmart), DJ-1 (ET1611-45, Hangzhou Huaan Biotechnology Co., LTD.) and MEF2A (A7911, ABclonal) were used. The immunoreactive proteins were incubated with the secondary antibodies (1:50,000; No. HA1001 for anti‑Rabbit IgG, Hangzhou Huaan Biotechnology Co., LTD.) and then exposed to SuperSignal West Dura Substrate (Thermo Scientific, MA, USA) with ECL (Biosharp, China).The protein expression level was normalized with the internal reference protein β‑actin.

#### Nuclear and cytoplasmic protein extraction

A Nuclear and Cytoplasmic Protein Extraction kit (P0027, Beyotime Biotechnology Co., Ltd., Shanghai, China.) was used to extract nuclear and cytoplasmic protein. The specific operation steps were carried out according to the kit instructions.

#### Quantitative real-time polymerase chain reaction (qRT–PCR)

Total RNA was extracted from the genioglossus tissues using RNA simple Total RNA Kit (TIANGEN, Beijing, China) and reverse-transcribed with a FastQuant RT Kit (Novoprotein, Shanghai, China) for the assay using SYBR Green Premix (Novoprotein, Shanghai, China). The primer sequences are shown in Table 2. The mRNA levels were normalized using GAPDH as a housekeeping gene and analyzed using the 2^−ΔΔCT^ method. The results were presented as means ± SD values.

#### MDA content determination

A malondialdehyde (MDA) content kit (Shanghai ZCI Biotechnology Co., LTD.) was used to measure MDA content. For every 0.1 g of tissue, 1 ml of extract was added for ice bath homogenization. We centrifuged 8000 g at 4 ℃ for 10 min, obtained the supernatant, and placed it on the ice to be measured. Exactly 0.6 ml of reagent was added into a 1.5 ml centrifuge tube with 0.2 ml sample and mixed thoroughly. We heated in a 95 ℃ water bath for 30 min, cooled in an ice bath, 10,000 g, 25 ℃, and centrifuged for 10 min. The supernatant was absorbed into a 1 ml glass colorimetric dish, and the absorbance at 532 nm and 600 nm was measured as A532 and A600, respectively, ΔA = A532–A600. MDA content was calculated according to sample mass (nmol/g) = [ΔA*V_T_ ÷ (Ɛ*d)*10^9^ ÷ (W*Vs ÷ V_TS_) = 51.6 × ΔA ÷ W. (W: Sample quality). Take same quality per sample, all sample MDA contents were detected and compared in the same time and the same conditions. MDA is the prototype of the so called thiobarbituric acid reactive substances (TBARS)^44^. MDA is one of the most frequently measured biomarkers of oxidative stress, namely of lipid peroxidation.

#### GSH-PX activity determination

A GSH-PX activity kit (Nanjingjiancheng Biotechnology Co., LTD.) was used to measure GSH-PX activity. Accurately weigh the tissue to be measured, add 9 times the volume of normal saline according to the ratio of weight (g) : volume (ml) = 1:9, homogenize at low temperature (0–4 ℃), 3500 RPM, centrifuge for 10 min, and take the supernatant to be measured (the concentration of the supernatant is measured with the BCA protein concentration detection kit). The specific operation steps were carried out according to the kit instructions. Enzymatic reaction was carried out first, enzyme tube test tube and non-enzyme tube were set for care, and then color reaction was carried out. After mixing, the absorbance of each tube was measured at 412 nm. GSH-PX activity was calculated according to the formula provided in the kit (Supplementary Figures).

### Statistical analysis

The data obtained in this experiment were statistically analyzed by SPSS 22.0 software and GraphPad Prism 9 Software for drawing. The data were normally distributed and the variance between the groups was homogeneous. In terms of mean ± standard deviation (SD). One-way analysis of variance was used for comparison between groups, with P < 0.05 representative difference was statistically significant.

### Institutional review board statement

The animal study protocol was approved by the Animal Care Committee of Changchun Weishi Testing Technology Service (approval number: 20220906-01), and all the animal care and experimental procedures were conducted in accordance with "The Guide for Care and Use of Laboratory Animals.

### Supplementary Information

Supplementary Figures.

## Data Availability

The datasets used and analyzed during the current study are available from the corresponding author on reasonable request.
