# Supplementary material for: Identifying key antioxidative stress factors regulating Nrf2 in the genioglossus with human umbilical cord mesenchymal stem-cell therapy
Source: Sci Rep. 2024 Mar 10;14:5838. doi: 10.1038/s41598-024-55103-8 (PMC10925593; doi:10.1038/s41598-024-55103-8)

Figure 1

NRF2


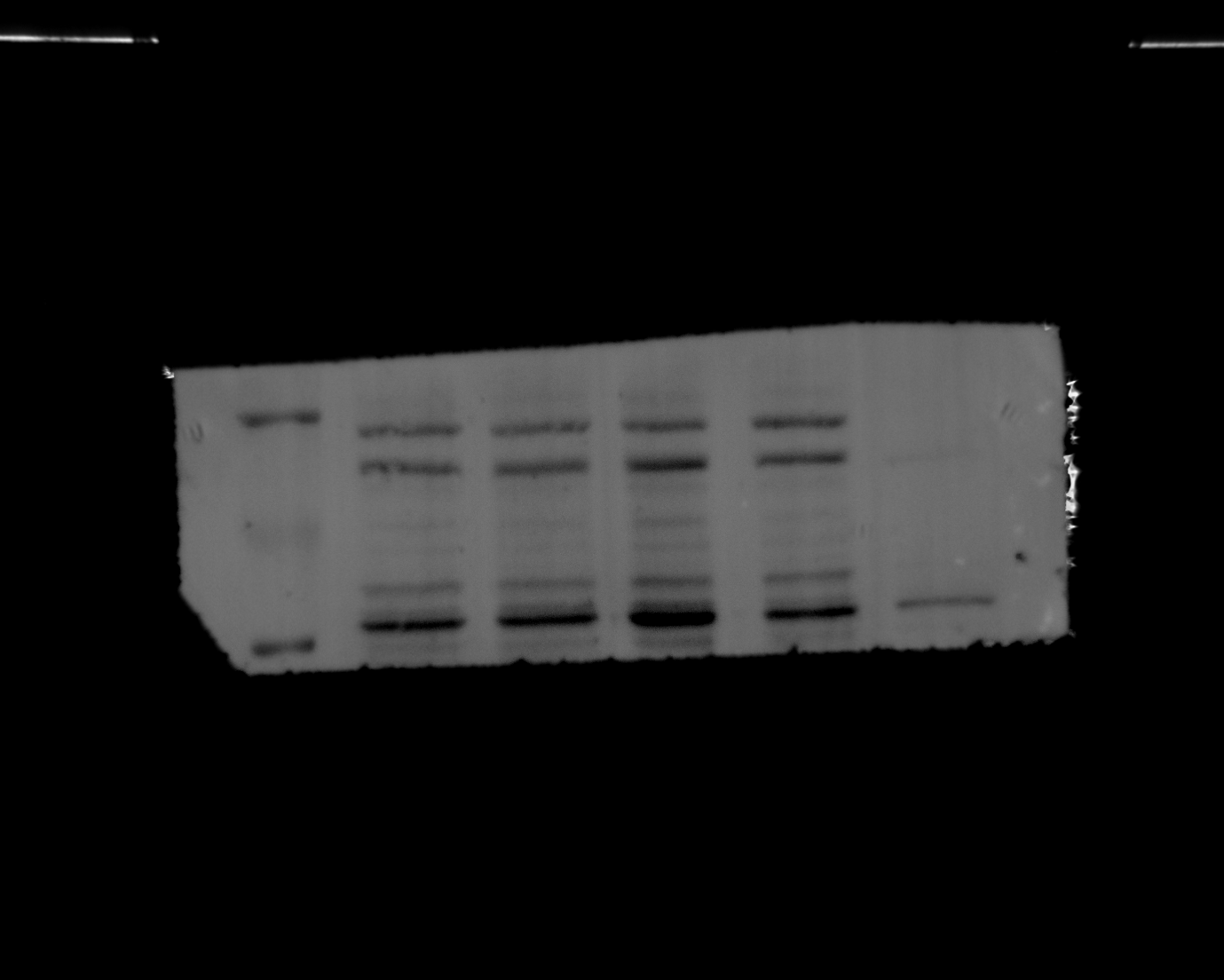


β-actin


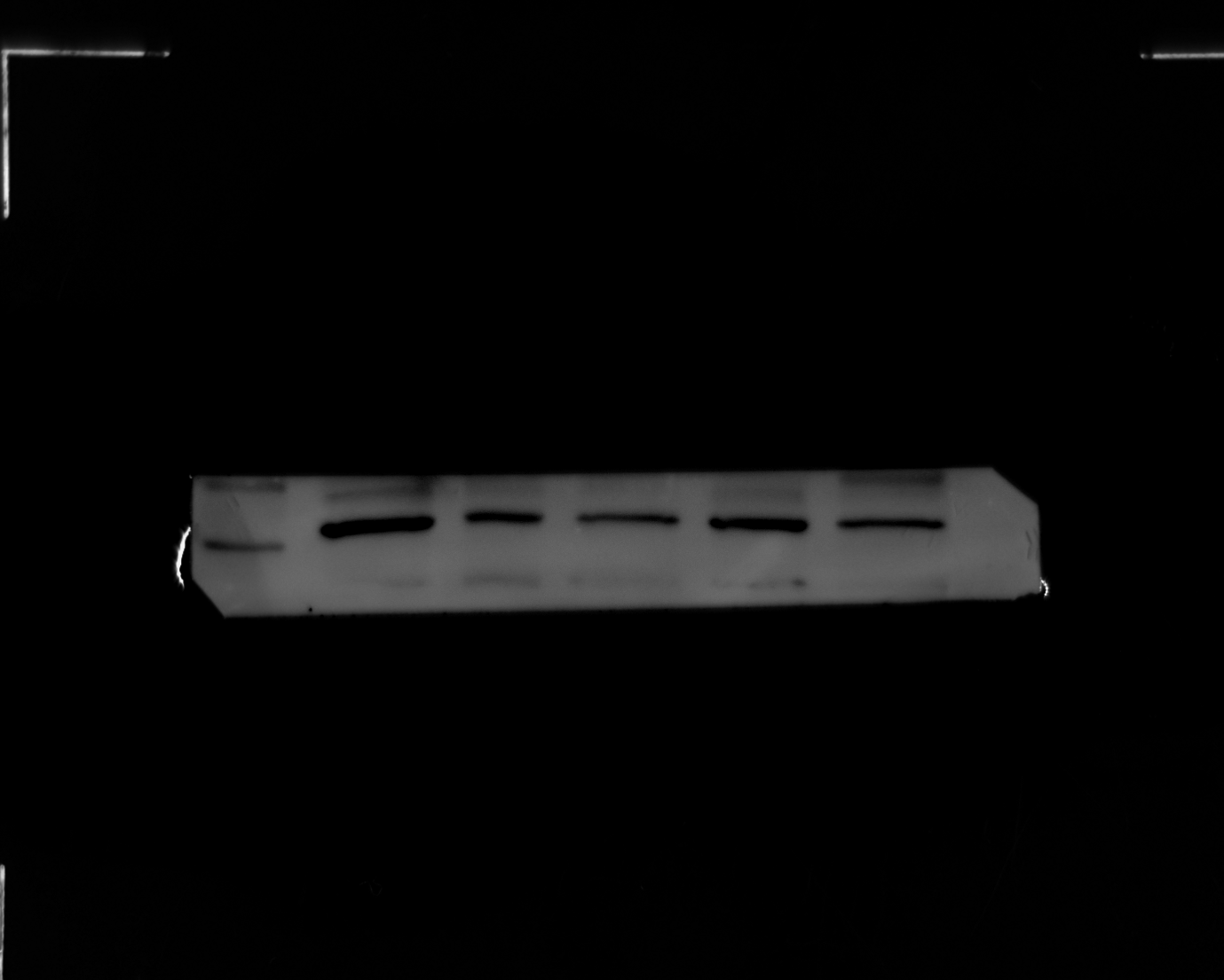


Figure 3

NRF2


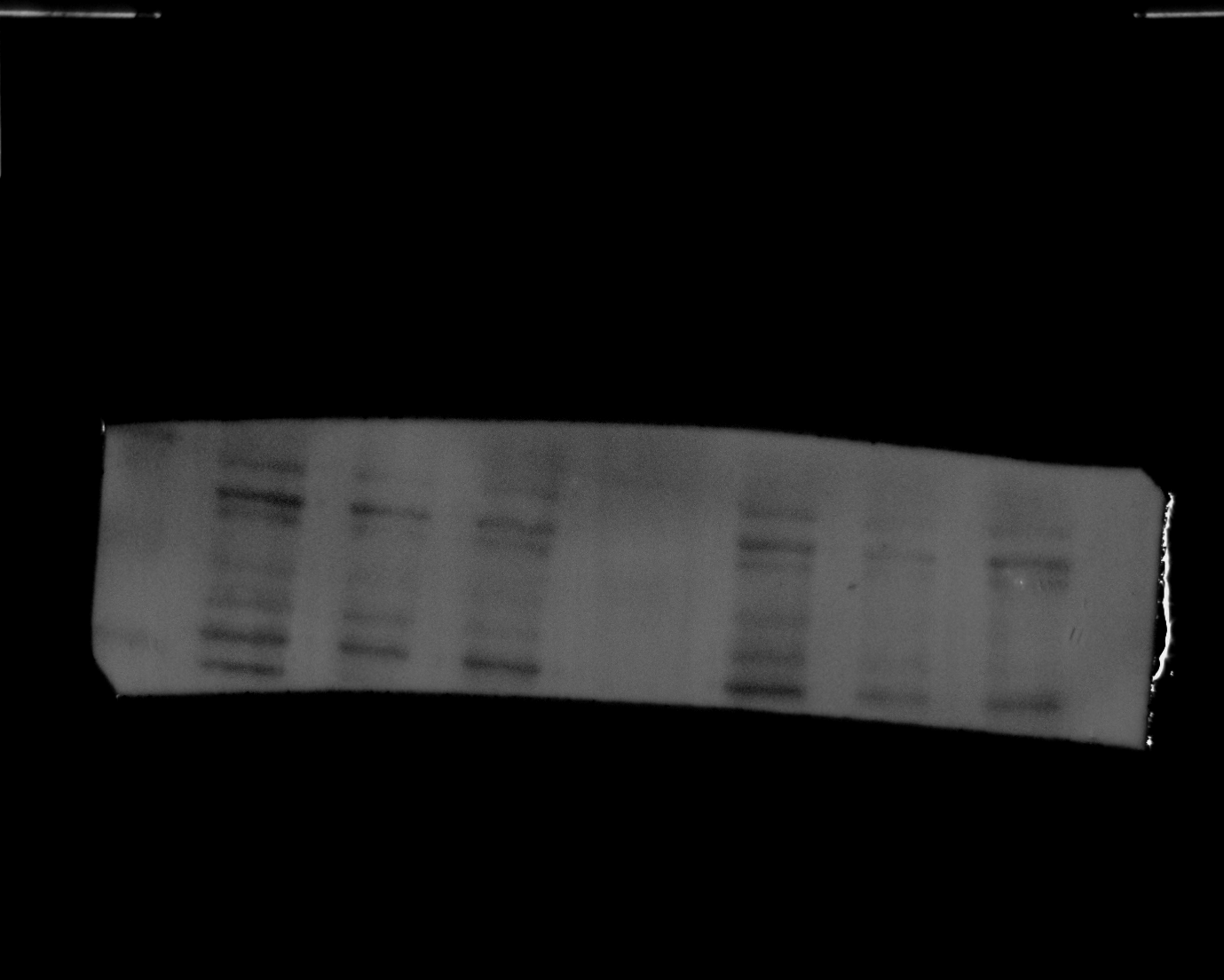


β-actin


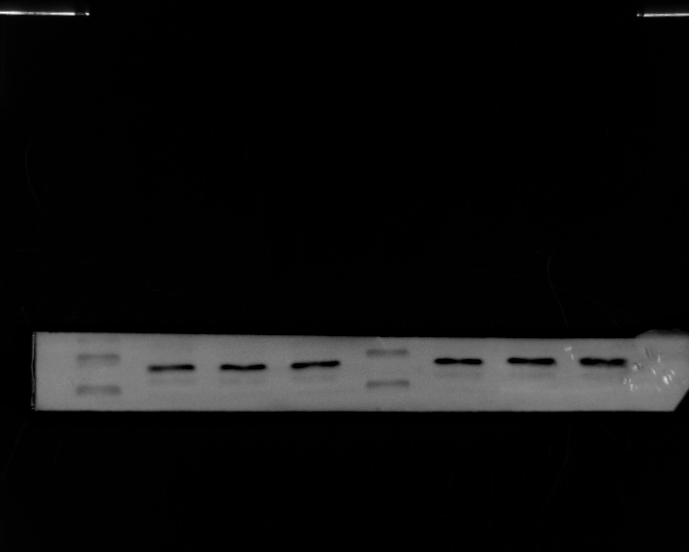


Figure 4

HO-1


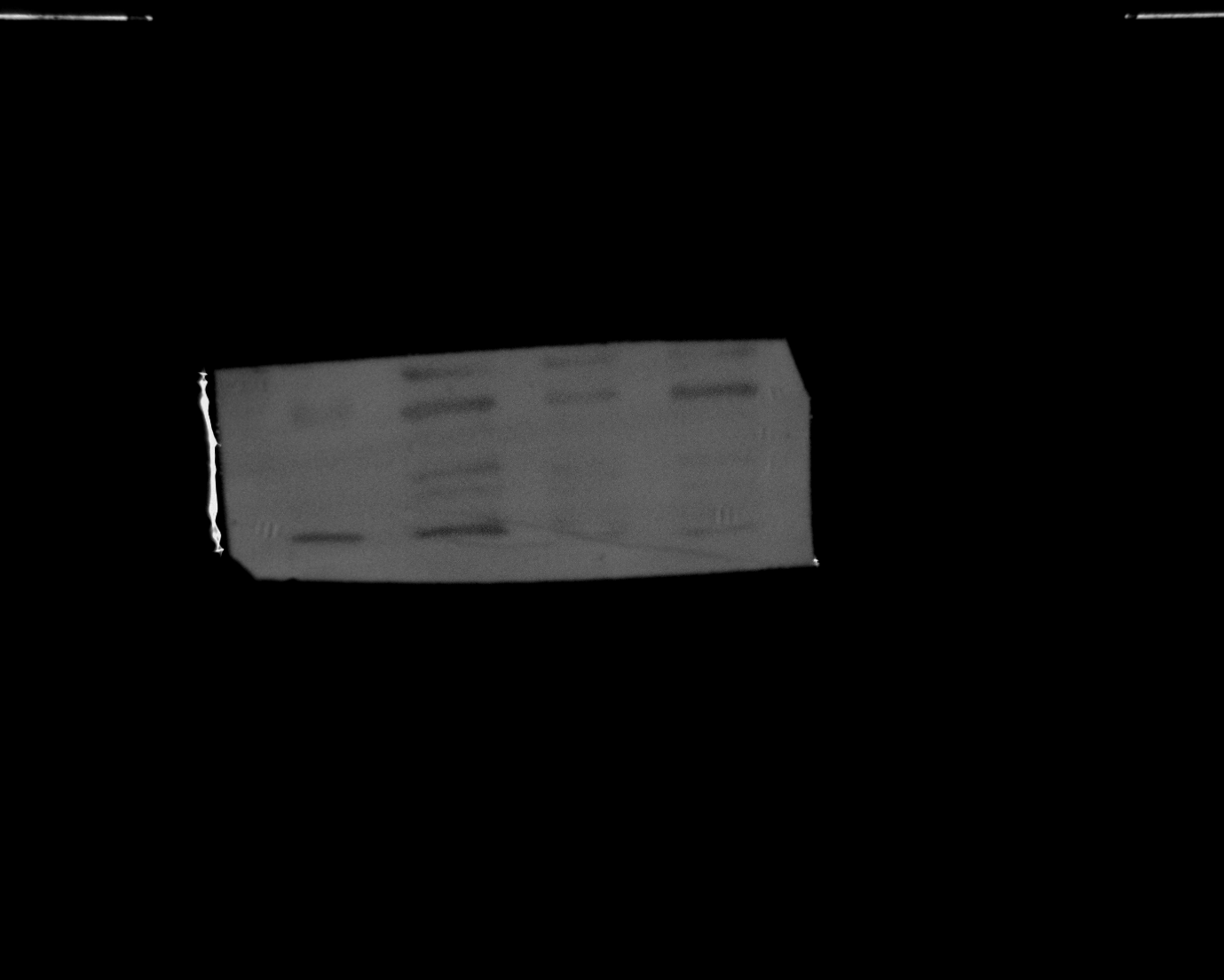


NQO1


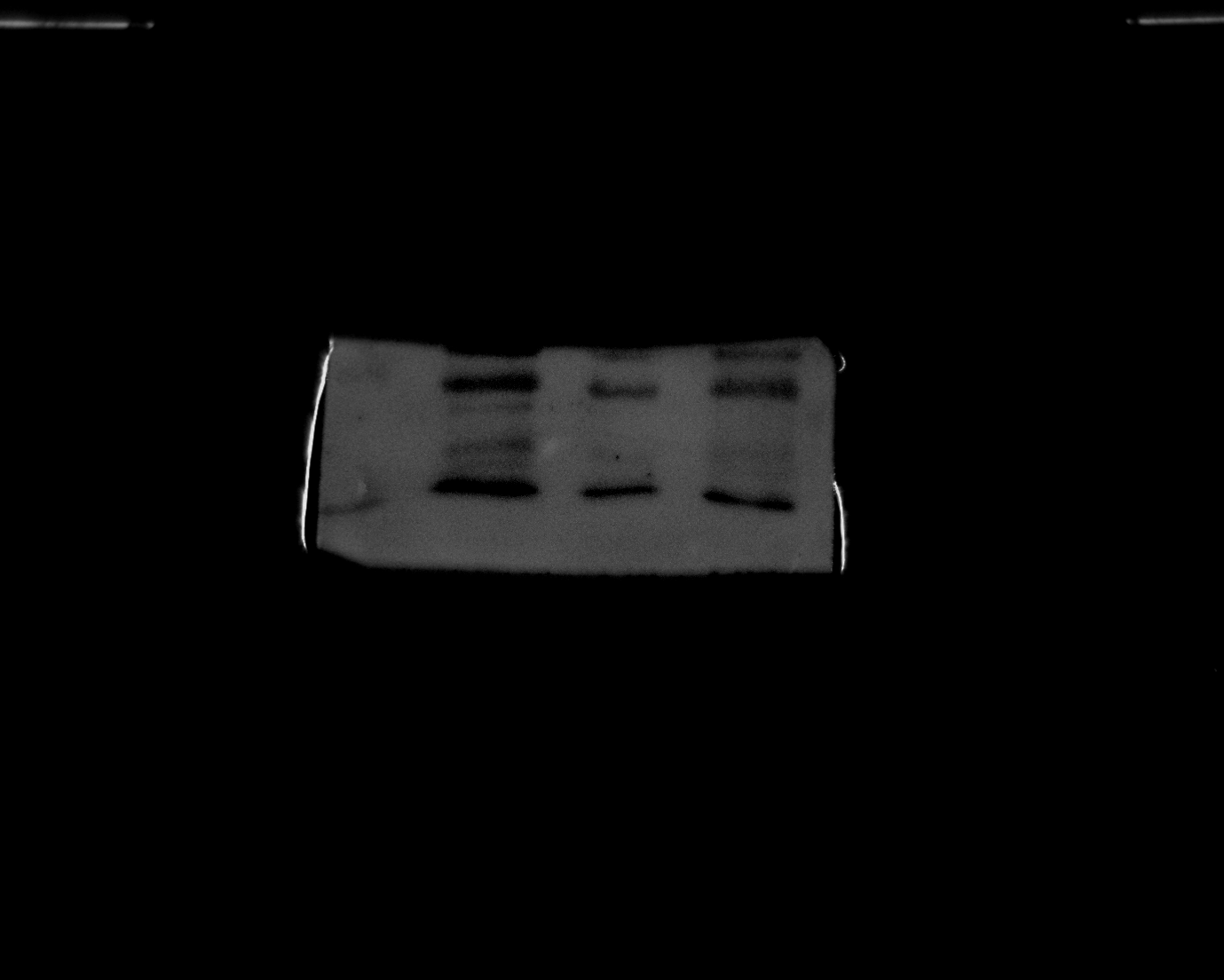


β-actin


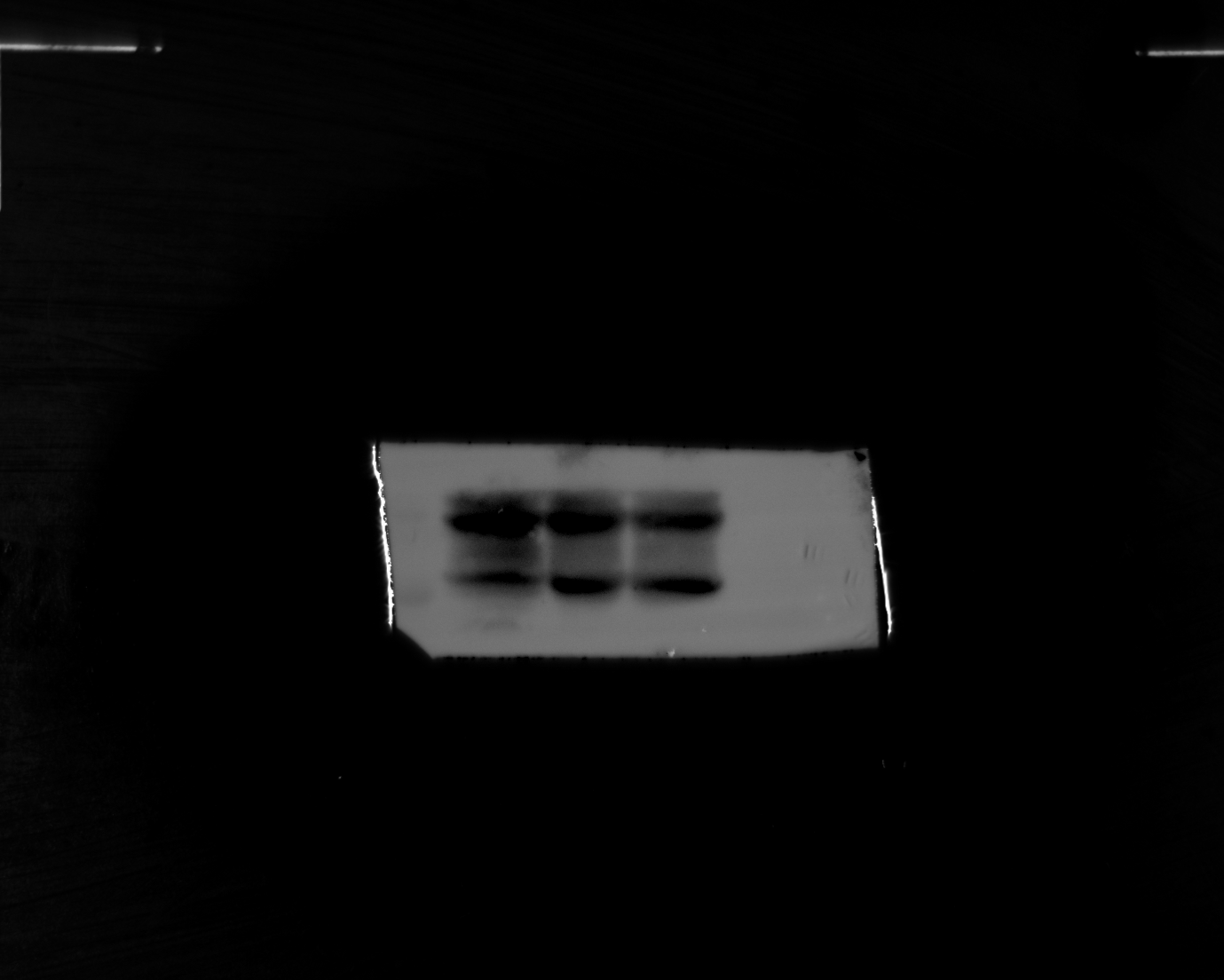


Figure 5

Nrf2


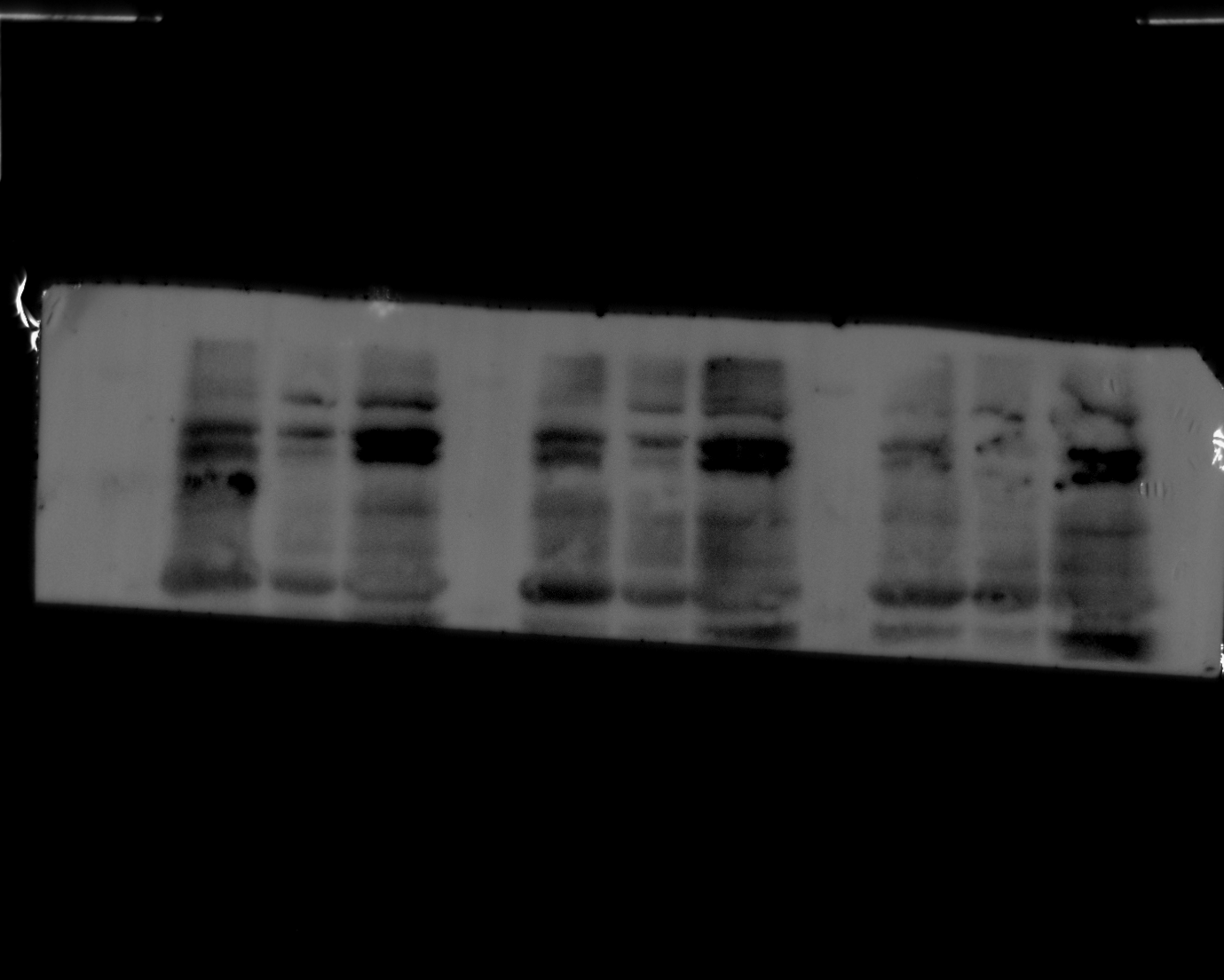


β-actin


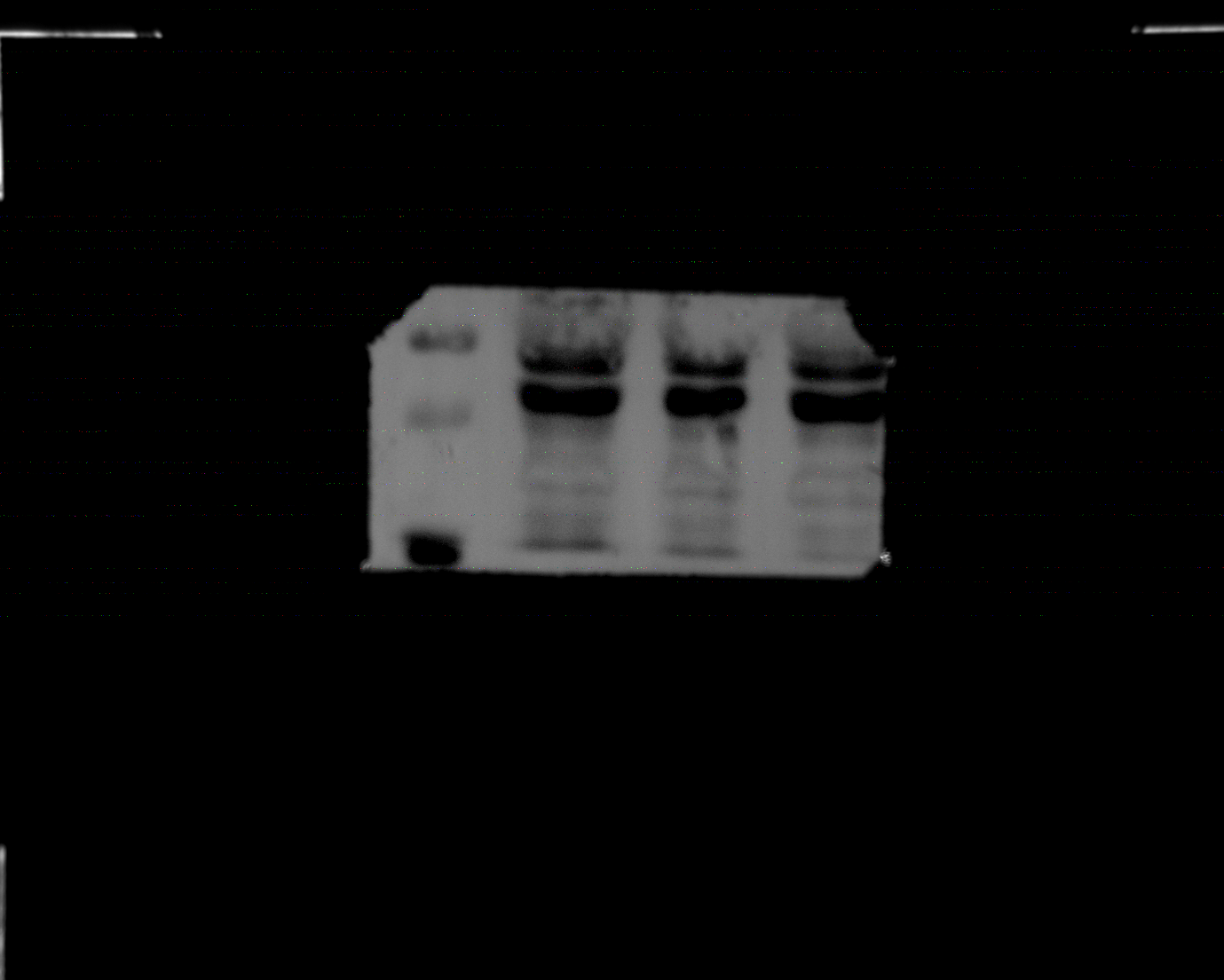

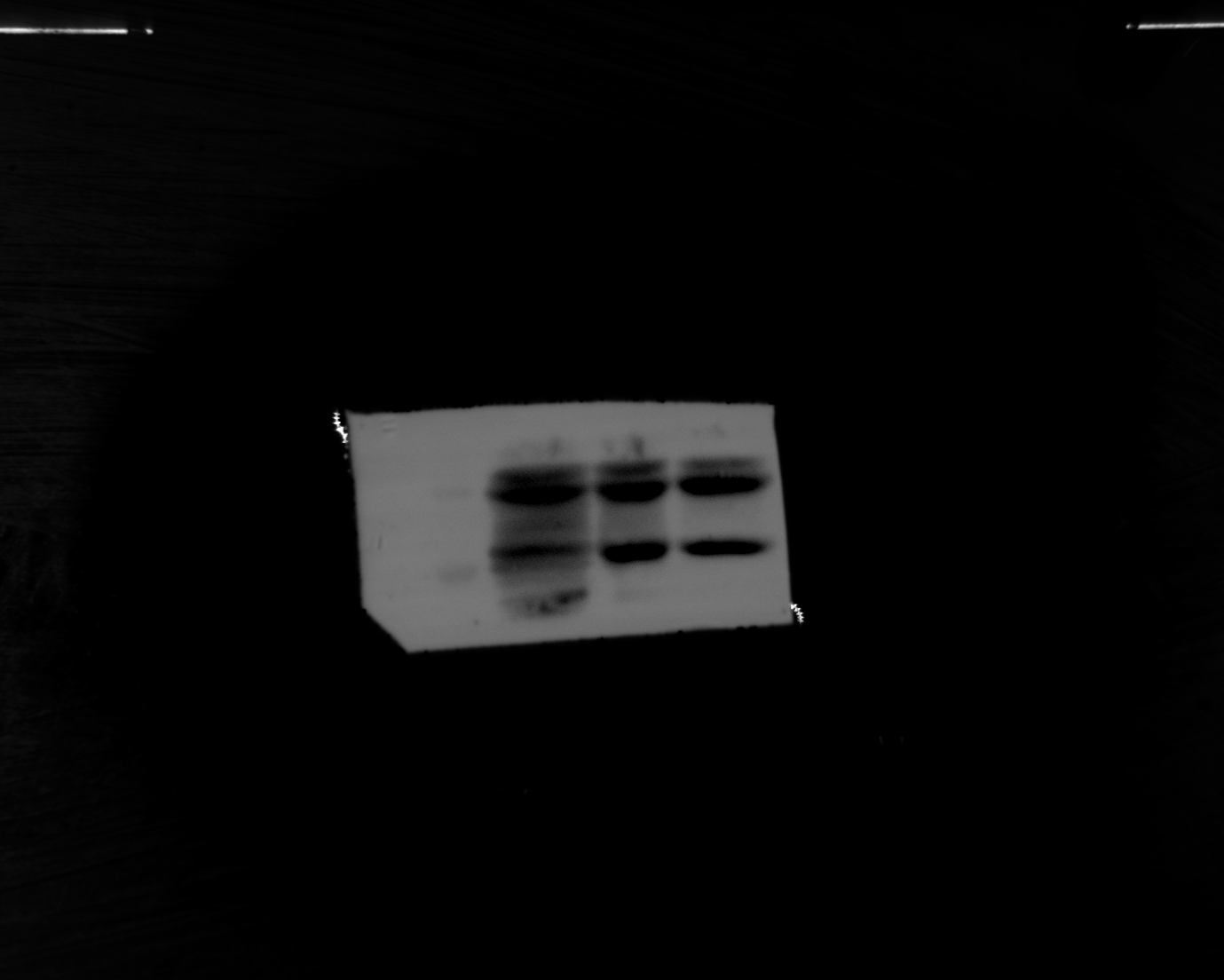


Figure 6

DJ-1


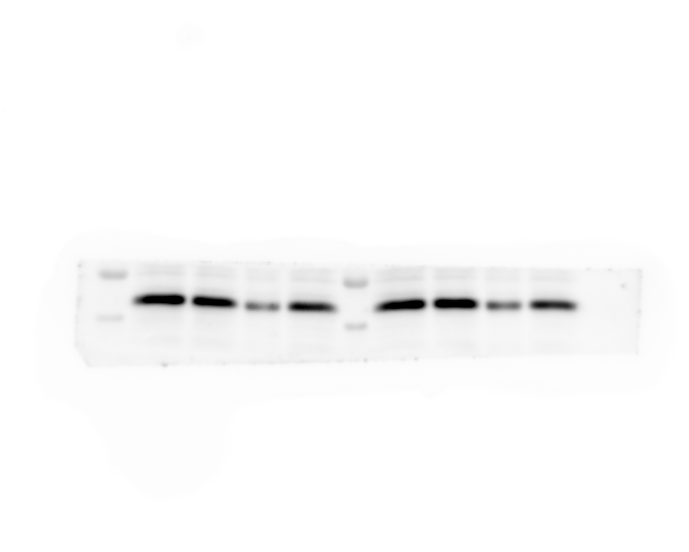


β-actin


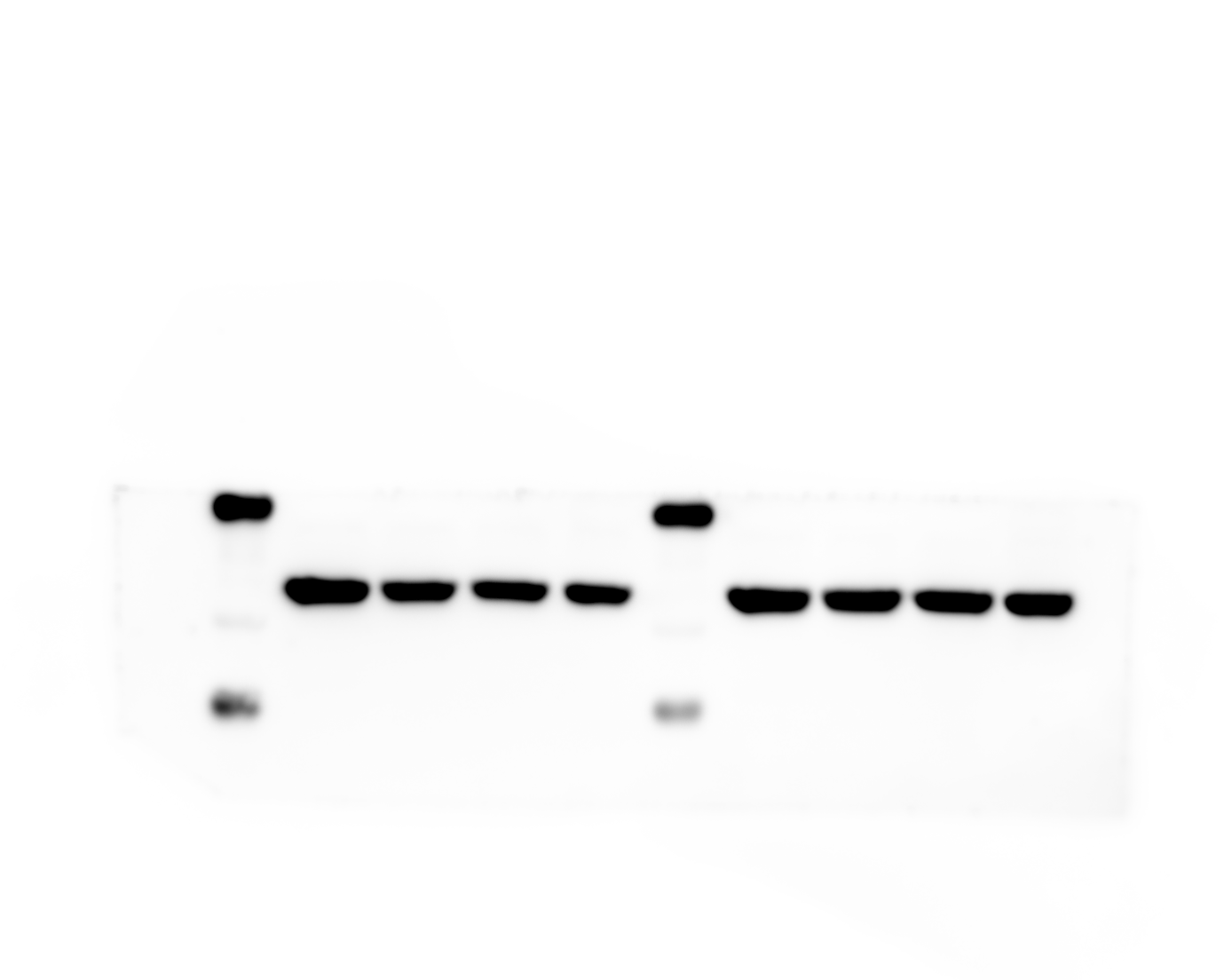


MEF2A


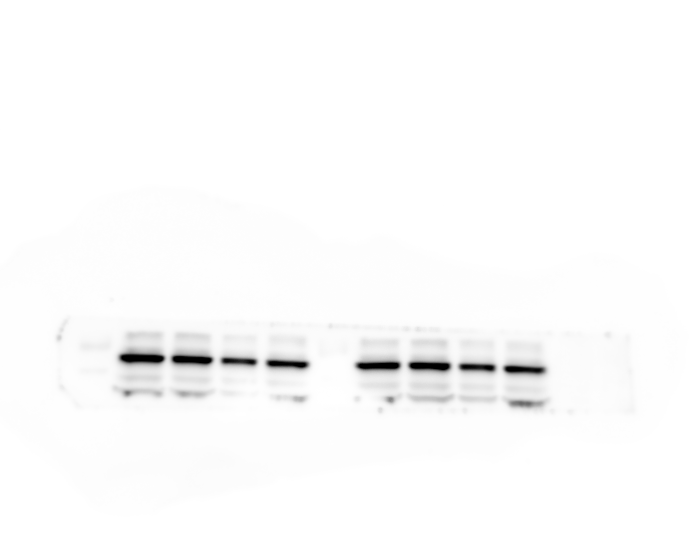


β-actin


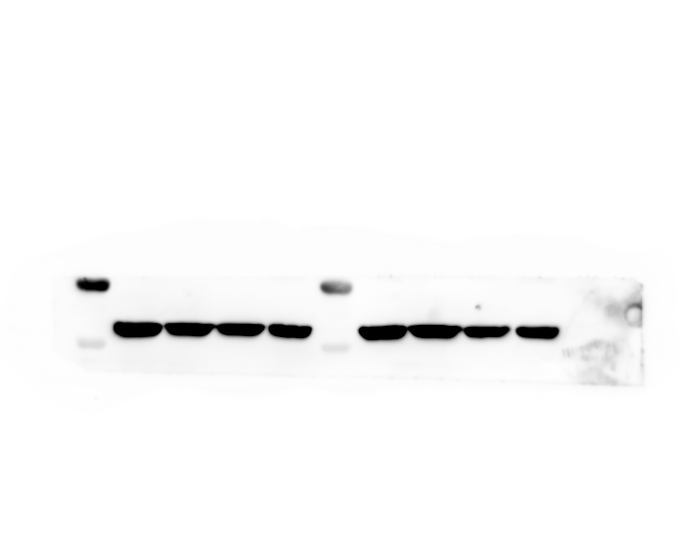

Supplement: Supplementary file 1 — Supplementary Figures. [file 41598_2024_55103_MOESM1_ESM.doc]
